# How does the association of general and central adiposity with glycaemia and blood pressure differ by gender and area of residence in a Malawian population: a cross-sectional study

**DOI:** 10.1093/ije/dyy047

**Published:** 2018-04-10

**Authors:** Kathleen Mudie, Debbie A Lawlor, Neil Pearce, Amelia Crampin, Laurie Tomlinson, Terence Tafatatha, Crispin Musicha, Dorothea Nitsch, Liam Smeeth, Moffat J Nyirenda

**Affiliations:** 1 Faculty of Epidemiology and Population Health, London School of Hygiene and Tropical Medicine, London, UK; 2 MRC Integrative Epidemiology Unit at the University of Bristol, Bristol, UK; 3 Population Health Sciences, Bristol Medical School, University of Bristol, Bristol, UK; 4 Malawi Epidemiology and Intervention Research Unit, Karonga, Malawi

**Keywords:** Diabetes, hypertension, blood pressure, blood glucose, body mass index, waist to hip ratio, obesity, sub-Saharan Africa

## Abstract

**Background:**

In high-income settings, body mass index (BMI) and measures of central adiposity, such as waist-to-hip ratio (WHR) are associated with cardiometabolic risk, but evidence from low-income settings, particularly sub-Saharan Africa (SSA), is limited. We assessed whether there are differences between central and general adiposity in their associations with fasting glucose, diabetes, systolic and diastolic blood pressures and hypertension, and whether these associations differ with gender or rural/urban setting in Malawi.

**Methods:**

We used data from a population-based study of 27 880 Malawian adults aged  ≥18 years, from both rural and urban areas. We used age-standardized z-scores of the means of BMI and WHR to directly compare their associations with glycaemic and blood pressure outcomes.

**Results:**

Mean fasting glucose and blood pressure values and odds of hypertension increased linearly across fifths of BMI and WHR, with stronger associations with BMI. For both BMI and WHR, the associations with outcomes were stronger in urban versus rural residents. The association with diabetes was stronger in women than men, whereas for blood-pressure related outcomes a stronger association was seen in men.

**Conclusions:**

BMI is more strongly associated with cardiometabolic risk in SSA, and might be a more useful measure than WHR, in this population. The greater positive association of adiposity with cardiometabolic outcomes in urban residents (where rates of overweight/obesity are already high) highlights the particular importance of addressing obesity within urban SSA populations.

**Key messages**

- BMI, a measure of general adiposity, is more strongly associated with glycaemia and blood pressures than measures of central adiposity in a large sub-Saharan African population.
- Positive associations of both general and central adiposity with glycaemia and blood pressures are stronger in urban compared with rural residents, even after adjustment for potential confounders.
- A stronger positive association with adverse cardio-metabolic health in urban areas, where rates of obesity and overweight are already high, emphasises the importance of obesity interventions within urban sub-Saharan African populations.

## Introduction

Non-communicable diseases (NCD) are replacing infectious diseases as the leading cause of adult death worldwide, including in low- and middle-income countries (LMIC). Being overweight or obese is a major modifiable risk factor for a wide range of NCDs including cardiovascular disease, type 2 diabetes mellitus and certain cancers.^1^ Body mass index (BMI) is the most commonly used measure for estimating general adiposity and is considered the most reliable proxy for body fat percentage in population health studies, in part because of ease of measurement.^2^

BMI does not distinguish between central or peripheral fat. Some evidence suggests that centrally distributed visceral fat and ectopic liver fat are associated with cardiometabolic outcomes independently of BMI or total fat mass.^3^ Among Western populations, waist circumference (WC), waist-to-hip ratio (WHR) and waist-to-height ratio (WHtR) are used as measures of central adiposity and have been shown to be positively associated with adverse cardiometabolic outcomes, with the strength of association similar to that of BMI.^1^^,^^4–6^

However, whether the same is true in sub-Saharan African (SSA) populations is unclear, because of the lack of evidence. The extent to which the marked lifestyle and dietary differences between rural and urban areas of SSA lead to differences in the associations between adiposity and cardiometabolic risk is largely unknown, but is relevant to being able to predict the emerging pattern of health and to develop appropriately tailored interventions aimed at preventing these outcomes.

We aimed to:

- determine whether there are differences between BMI and waist-based (central adiposity) measurements in their associations with fasting glucose, diabetes, systolic and diastolic blood pressures (SBP and DBP) and hypertension in an SSA population;
- determine whether any of these associations of BMI and central adiposity with glycaemic or BP outcomes differ between gender or rural/urban residence.

## Methods

### Study design, setting and participants

Cross-sectional data from participants in the Malawi Epidemiology and Intervention Research Unit (MEIRU) NCD study was used. The methods used to recruit and collect data from these participants were described in detail previously.^7^ Briefly, data collection began in June 2013, and includes a rural and an urban site. The rural area is an established research site—the Health and Demographic Surveillance System (HDSS) in southern Karonga (Northern region)—with a typical rural population.^8^ An urban area (Area 25) of Lilongwe, the capital city of Malawi (Central region), was selected because it included a broad spectrum of socioeconomic groups in a high-density area where the Ministry of Health (MoH) was establishing a chronic care clinic, similar to that in Karonga, to which we could link our study participants.^9^

**Figure 1:**
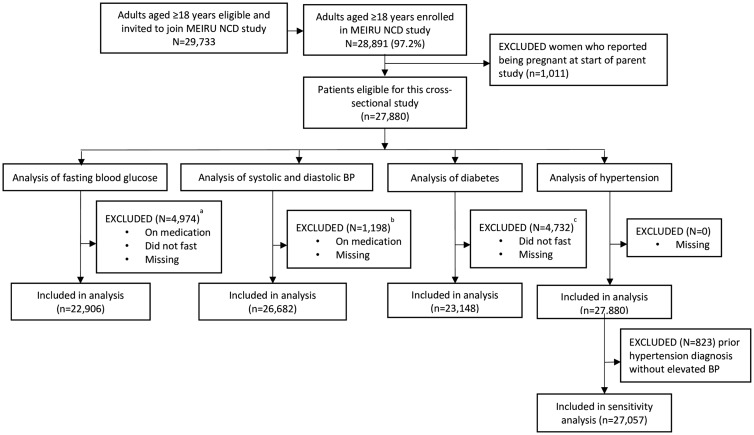
STROBE flow chart of participant inclusion into each outcome analysis; ^a^21 participants had more than one exclusion criterion; the majority of excluded participants had missing glucose values (*N* = 4726). ^b^19 participants had more than one exclusion criterion; the majority of excluded participants were on medication (*N* = 1184). ^c^Six participants had more than one exclusion criterion; the majority of excluded participants were missing diabetes values (*N* = 4693).

All adults aged 18 years and older, who were usually resident at a household in either study area and were able to consent, were eligible and invited to be included in the study; 29 733 were contacted and 28 891 (97%) were recruited. For this study, we excluded a priori women who self-reported being pregnant (*N* = 1011) and, for analyses with fasting glucose and BP treated as continuous measurements, we excluded those on antidiabetic (*N* = 225) or on antihypertensive (*N *= 1184) medication, respectively. We also excluded from the analyses of glucose-related outcomes those who reported not fasting (*N* = 44) and those with missing glucose data (*N* = 4726). Following these exclusions, samples ranged from 22 906 to 27 880 for different outcomes (Figure 1).

All participants provided informed written consent and the study was approved by the Malawi National Health Sciences Research Committee (NHSRC; protocol #1072) and London School of Hygiene and Tropical Medicine (LSHTM) Ethics Committee (protocol #6303).^10^

### Outcome and exposure variables

All questionnaires and measurements (anthropometric and BP) were collected by trained field workers using standardized protocols (see Supplementary materials for more details of the standard protocols, available as Supplementary data at *IJE* online). Blood samples were taken by phlebotomy-trained nurses. Electronic data entry (using tablets) at the point of collection, with pre-programmed ranges and internal consistency checks, was used. In interviews, participants were asked about any medications or previous diagnoses.

Hypertension was defined as having either an SBP  ≥140 mmHg or a DBP  ≥90 mmHg or being on antihypertensive medication.^10^ Participants who self-reported a previous diagnosis of hypertension (but did not have elevated BP in our assessments) were treated as if they did not have hypertension, because it is common for the term to be used by health care professionals on the basis of a single BP measurement. In sensitivity analyses, we explored whether this affected our results by removing these participants from analyses.

Participants were asked in interviews whether they had previously been diagnosed with diabetes and about use of any regular medication. Diabetes was defined as having any of the following: fasting glucose  ≥7.0 mmol/l, currently taking antidiabetic medication or a self-report of a previous diabetes diagnosis.^10^ We assumed that all diabetic participants have type 2 diabetes mellitus because it is accountable for over 90% of cases in SSA and because we do not know at what age the few patients on insulin were diagnosed or were started on treatment.^11^^,^^12^

### Potential confounders

We considered smoking status, alcohol intake, physical activity and socioeconomic position (SEP) to be potential confounders for the associations between adiposity measurements and the various outcomes. Potential confounders were selected a priori, based on existing evidence regarding the relations to both adiposity and our outcomes. In response to peer reviewer comments, we additionally adjusted for the number of live births in women. Data on these variables were collected using a modified version of the World Health Organization (WHO) STEPS questionnaire, by fieldworkers who were fluent in local African languages (as well as English).^7^ The methods used to measure and categorize these variables can be found in the Supplementary materials (available as Supplementary data at *IJE* online).

### Statistical analyses

All analyses were carried out using STATA^TM^ version 14.0 (StataCorp, TX, USA). In order to directly compare the directions and magnitudes of the associations of BMI, WC, WHR and WHtR, internally age-standardized z-scores of the means were used for each anthropometric measure.

We considered a priori that WHR would be the best measure of health adiposity distribution, as both a smaller waist (less central adiposity) and larger hips (greater peripheral adiposity) have been associated with better cardiometabolic health.^2^^,^^4^ We also assumed that WHR would be the measure of central adiposity that was least correlated with (and therefore distinct from) BMI. We tested this by examining pairwise partial correlation coefficients for zBMI, zWHR, zWC and zWHtR in the overall cohort and stratified by gender and area of residence.

To explore whether associations were linear or not, we first plotted means (for continuous outcomes) and percentages (for binary outcomes) against z-score fifths of BMI and WHR. Multiple linear (continuous outcomes) and logistic (binary outcomes) regression was used to further explore the associations. We examined the pattern of association by inspecting the graphs and by running regression analyses with the z-score fifths of BMI/WHR entered into models as four indicator variables (five categories) and as a continuous score of the fifths. We compared the two models and considered associations to be non-linear if graphs were monotonic; the linear trend *P*-value was >0.05 (two-sided) and *P*-value for the comparison of the two models <0.05 (two-sided). Per-fifth associations were presented if there was no strong evidence for a non-linear effect between zBMI/zWHR with each outcome.

We then adjusted for potential confounding by smoking status, alcohol intake, physical activity and SEP (wealth). All regression analyses were undertaken in the overall cohort and then stratified into four groups: rural women, rural men, urban women and urban men. Evidence for differences in associations between these four groups was explored by examining the stratified results and by testing for interactions between either gender or area of residence and BMI/WHR (as a continuous score of z-score fifths) in their associations with each outcome, using a likelihood ratio test.

In additional sensitivity analyses, we repeated the main analyses with BMI and WHR age-standardized z-scores determined within the four area of residence and gender strata (rather than within the overall cohort), to explore whether any marked differences in age distribution between these groups might have influenced our results. We also repeated the main analyses for the associations of zWC and zWHtR with each outcome, using the same analytical approach as described above for zBMI/zWHR.

## Results

The distributions of study characteristics, including missing data for each variable, for the overall cohort and by gender and area of residence are shown in Table 1. Urban residents were younger and more likely to be in the highest fifth of wealth. Smoking was rare, and 93% of the overall cohort reported never smoking; it was more common in urban populations. Most participants reported no alcohol intake in the past year, but there were marked gender differences with few women drinking alcohol and ∼50% of men drinking at least some in the past year. The vast majority of all participants (86%) reported high levels of physical activity, particularly rural women. Of the cohort: 13% were hypertensive, with levels highest in urban men; 2% were diabetic with levels highest in urban residents; BMI was higher in women than men and in urban than rural residents; And WHR varied less between groups and was lowest in urban women.

**Table 1.** Characteristics of the whole study population and in women and men, rural and urban resident Malawian adults

			Rural Residents		Urban Residents	
		Total *N* = 27 880 (%)	Females *n* = 7528 (27%)	Males *n* = 5849 (21%)	Females *n *= 9290 (33%)	Males *n* = 5213 (19%)
Sex *N* (%)	Women	16 818 (60.3)	–	–	–	–
	Men	11 062 (39.7)	–	–	–	–
Residence *N* (%)	Rural	13 377 (48.0)	–	–	–	–
	Urban	14 503 (52.0)	–	–	–	–
Age	Mean (±SD)	35.9 (15.1)	39.4 (16.5)	38.3 (16.5)	33.0 (12.6)	33.5 (13.8)
Wealth fifths^a^*N* (%)	1 (<£102)	4988 (17.9)	1960 (26.0)	1425 (24.4)	1073 (11.5)	530 (10.2)
	2 (£102-£158)	5998 (21.5)	2388 (31.7)	1918 (32.8)	1107 (11.9)	585 (11.2)
	3 (£158-£307)	5346 (19.2)	1483 (19.7)	1163 (19.9)	1773 (19.1)	927 (17.8)
	4 (£307-£583)	6468 (23.2)	1193 (15.8)	952 (16.3)	2772 (29.8)	1551 (29.7)
	5 (>£583)	4851 (17.4)	504 (6.7)	391 (6.7)	2435 (26.2)	1521 (29.2)
	Missing	229 (0.8)	0 (0)	0 (0)	130 (1.4)	99 (1.9)
Smoking status *N* (%)	Never	26 583 (95.3)	7504 (99.7)	4798 (82.0)	9230 (99.3)	4339 (83.2)
	Ex-smoker	712 (2.5)	10 (0.1)	262 (4.5)	38 (0.4)	402 (7.7)
	Light (<6 cigs/day)	810 (2.9)	12 (0.2)	485 (98.3)	15 (0.2)	298 (5.7)
	Heavy (≥6 cigs/day)	487 (1.7)	2 (0.03)	304 (5.2)	7 (0.1)	174 (3.3)
Alcohol intake * N* (%)	None in past year	22 627 (81.2)	7251 (96.3)	3394 (58.0)	8709 (93.7)	3273 (62.8)
	Light (<8 units/wk)	4015 (14.4)	266 (3.5)	1966 (33.6)	510 (5.5)	1273 (24.4)
	Moderate (8-21 units/wk)	990 (3.5)	11 (0.1)	386 (6.6)	64 (0.7)	529 (10.2)
	Heavy (>21 units/wk)	248 (0.9)	0 (0.0)	103 (1.8)	7 (0.1)	138 (2.6)
Physical activity level *N* (%)	Low (<4 METs/day)	1044 (3.7)	182 (2.4)	245 (4.2)	186 (2.0)	431 (8.3)
	Moderate (4-7 METs/day)	2831 (10.2)	348 (4.6)	765 (13.1)	596 (6.4)	1122 (21.5)
	High (>7 METs/day)	24 005 (86.1)	6998 (93.0)	4839 (82.7)	8508 (91.6)	3660 (70.2)
Previous hypertension diagnosis *N* (%)	No	25 354 (90.9)	6879 (91.4)	5624 (96.1)	8047 (86.6)	4804 (92.1)
	Yes, on medication	1184 (4.2)	380 (5.0)	127 (2.2)	497 (5.4)	180 (3.5)
	Yes, not on medication	1342 (4.8)	269 (3.6)	98 (1.7)	746 (8.0)	229 (4.4)
Systolic BP *N* (%)	Mean (±SD)	122.8 (17.6)	119.6 (19.3)	124.3 (15.6)	121.7 (17.6)	127.8 (15.7)
	Missing	20 (0.07)	8 (0.1)	2 (0.03)	8 (0.1)	2 (0.04)
Diastolic BP * N* (%)	Mean (±SD)	73.6 (10.8)	73.1 (10.5)	73.0 (10.3)	74.2 (11.0)	74.0 (11.5)
	Missing	19 (0.07)	8 (0.1)	2 (0.03)	7 (0.1)	2 (0.04)
Hypertensive *N* (%)	No	23 790 (85.3)	6430 (85.4)	5060 (86.5)	7947 (85.5)	4353 (83.5)
	Yes	4090 (14.7)	1098 (14.6)	789 (13.5)	1343 (14.5)	860 (16.5)
Previous diabetes diagnosis	No	27 552 (98.8)	7460 (99.1)	5808 (99.3)	9155 (98.5)	5129 (98.4)
	Yes, on medication	225 (0.8)	46 (0.6)	33 (0.6)	91 (1.0)	55 (1.1)
	Yes, not on medication	103 (0.4)	22 (0.3)	8 (0.1)	44 (0.5)	29 (0.6)
Fasting blood glucose *N* (%)	Mean (±SD)	4.8 (1.3)	4.7 (1.2)	4.7 (1.2)	4.8 (1.3)	4.8 (1.3)
	Missing	4726 (16.9)	823 (10.9)	844 (14.4)	1777 (19.1)	1282 (24.6)
Diabetic *N* (%)	No	22 634 (81.2)	6588 (87.5)	4925 (84.2)	7307 (78.6)	3814 (73.2)
	Yes	558 (2.0)	123 (1.6)	84 (1.4)	219 (2.4)	132 (2.5)
	Missing	4688 (16.8)	817 (10.9)	840 (14.4)	1764 (19.0)	1267 (24.3)
Weight *N* (%)	Median (IQR)	58.2 (52.0, 66.1)	54.4 (48.7, 61.8)	57.8 (53.0, 63.6)	59.8 (52.5, 70.4)	60.9 (55.6, 68.0)
	Missing	34 (0.1)	15 (0.2)	13 (0.2)	5 (0.1)	1 (0.02)
BMI *N* (%)	Median (IQR)	22.4 (20.4, 25.4)	22.6 (20.4, 25.3)	21.2 (19.8, 22.9)	24.3 (21.5, 28.3)	21.7 (20.1, 24.0)
	Missing	56 (0.2)	31 (0.4)	15 (0.3)	8 (0.1)	2 (0.04)
WC *N* (%)	Median (IQR)	77.3 (72.3, 85.0)	79.1 (73.6, 86.4)	76.1 (72.5, 81.0)	78.0 (71.5, 87.2)	76.0 (72.0, 83.0)
	Missing	35 (0.1)	18 (0.2)	10 (0.2)	6 (0.1)	1 (0.02)
WHR *N* (%)	Median (IQR)	0.84 (0.80, 0.88)	0.85 (0.81, 0.90)	0.85 (0.82, 0.89)	0.80 (0.76, 0.85)	0.84 (0.80, 0.88)
	Missing	43 (0.1)	21 (0.3)	12 (0.2)	9 (0.1)	1 (0.02)
WHtR *N* (%)	Median (IQR)	0.48 (0.45, 0.54)	0.51 (0.47, 0.56)	0.46 (0.44, 0.49)	0.50 (0.46, 0.56)	0.46 (0.43, 0.50)
	Missing	59 (0.2)	33 (0.4)	15 (0.3)	9 (0.1)	2 (0.04)

Cigs, cigarettes; wk, week; MET, metabolic equivalent; IQR, interquartile range.

a Equivalent values in Malawian Kwacha are <97 000MWK, 97 000–150 500MWK, 150 500–291 500MWK, 291 500–553 500MWK and >553 500 for each fifth.

Among the participants, 17% did not have a fasting glucose measure; levels of missing data were <1% for all other variables. Thus, the numbers included in analyses with fasting glucose/diabetes as outcomes are smaller than those with BP/hypertension as outcomes (Figure 1). A missing fasting glucose measure was more likely if participants were male, urban residents, ex-smokers, heavy consumers of alcohol, less physically active and in the lowest BMI fifths (Supplementary Table 2, available as Supplementary data at *IJE* online). For most of these differences, the magnitudes were small.

In the overall cohort, zWHR was only weakly correlated with zBMI (partial correlation = 0.15), whereas zWC and zWHtR were strongly correlated with zBMI (Table 2). When examined in the four strata, the patterns were broadly similar.

**Table 2.** Age-adjusted partial correlations of anthropometric measurements

	zBMI	zWC	zWHR	zWHtR
Whole cohort *N* = 27 880				
zBMI	1			
zWC	0.83	1		
zWHR	0.15	0.53	1	
zWHtR	0.84	0.92	0.49	1
Rural females *N* = 7528				
zBMI	1			
zWC	0.85	1		
zWHR	0.24	0.6	1	
zWHtR	0.84	0.95	0.63	1
Rural males *N* = 5849				
zBMI	1			
zWC	0.82	1		
zWHR	0.31	0.58	1	
zWHtR	0.83	0.89	0.62	1
Urban females *N* = 9290				
zBMI	1			
zWC	0.86	1		
zWHR	0.26	0.58	1	
zWHtR	0.86	0.96	0.61	1
Urban males *N* = 5213				
zBMI	1			
zWC	0.79	1		
zWHR	0.33	0.65	1	
zWHtR	0.81	0.92	0.67	1

zBMI, z-score, age-standardized body mass index; zWC, z-score, age-standardized waist-circumference; zWHR, z-score, age-standardized waist-to-hip ratio; zWHtR, z-score, age-standardized waist-to-height ratio.

### Differences between BMI and WHR in their associations with glycaemic and BP outcomes

**Figure 2:**
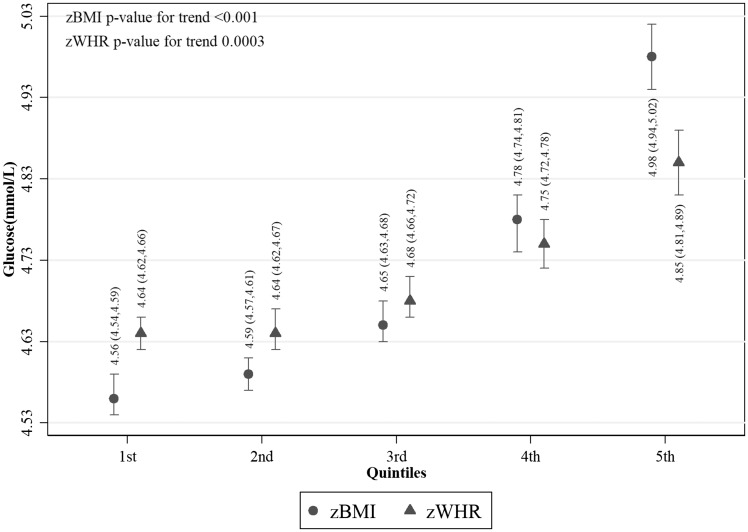
Mean fasting glucose per fifth of zBMI and zWHR in participants without diabetes (*N* = 22 906); zBMI, z-score age-standardized body mass index; zWHR, z-score age-standardized waist-to-hip ratio. Dots/triangles and first numerical results are mean fasting glucose (mmol/l); ends of vertical lines and numerical results in brackets represent the 95% confidence interval of the percentages.

**Figure 3:**
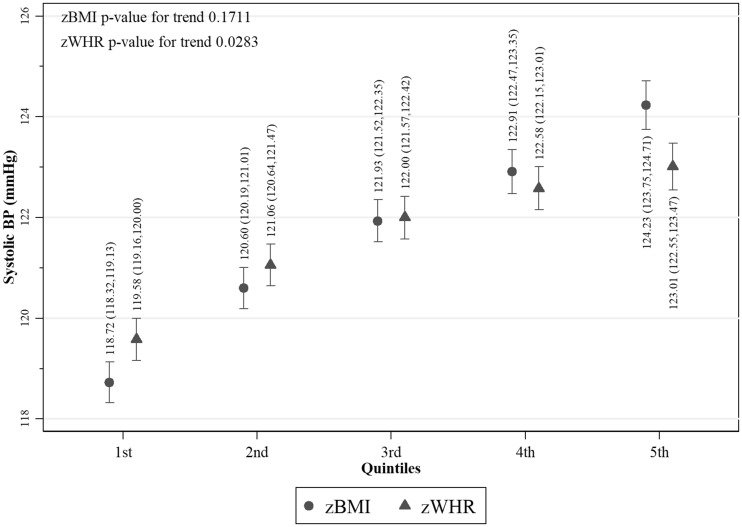
Mean systolic blood pressure per fifth of zBMI and zWHR in participants without hypertension (*N* = 26 682); zBMI, z-score age-standardized body mass index; zWHR, z-score age-standardized waist-to-hip ratio. Dots/triangles and first numerical results are mean systolic blood pressure (mmHg); ends of vertical lines and numerical results in brackets represent the 95% confidence interval of the percentages.

**Figure 4:**
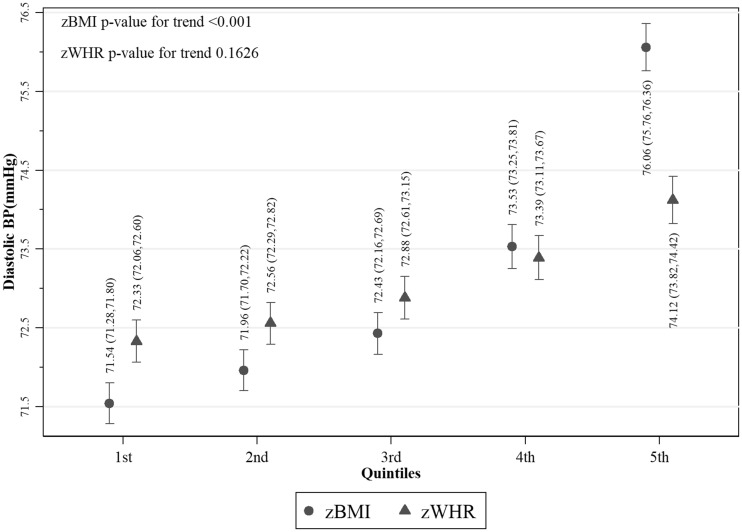
Mean diastolic blood pressure per fifth of zBMI and zWHR in participants without hypertension (*N* = 26 683); zBMI, z-score age-standardized body mass index; zWHR, z-score age-standardized waist-to-hip ratio. Dots/triangles and first numerical results are mean diastolic blood pressure (mmHg); ends of vertical lines and numerical results in brackets represent the 95% confidence interval of the percentages.

**Figure 5:**
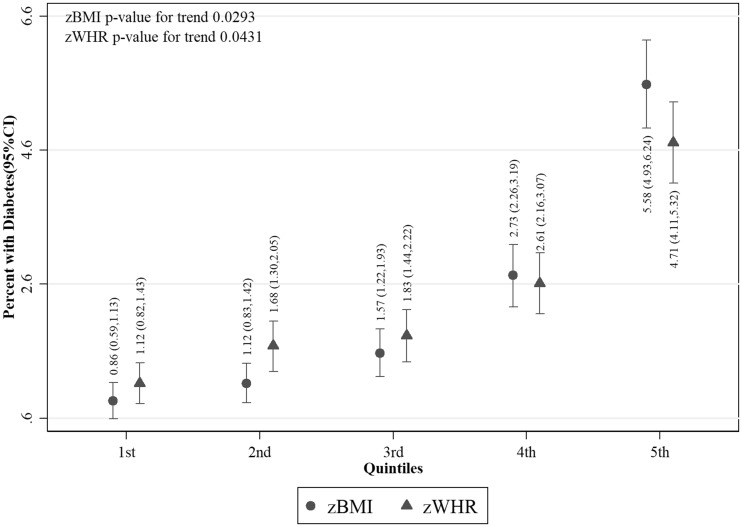
Percentage with diabetes per fifth of zBMI and zWHR (*N* = 23 148); zBMI, z-score age-standardized body mass index; zWHR, z-score age-standardized waist-to-hip ratio. Dots/triangles and first numerical results are percentage with diabetes; ends of vertical lines and numerical results in brackets represent the 95% confidence interval of the percentages. Diabetes was defined as having any of the following: fasting glucose ≥7.0 mmol/l, currently taking antidiabetic medication or a self-report of a previous diabetes diagnosis.

**Figure 6:**
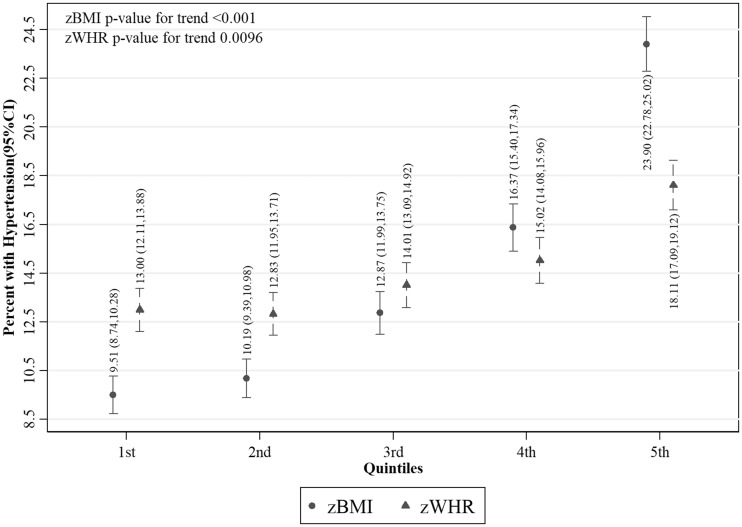
Percentage with hypertension per fifth of zBMI and zWHR (*N* = 27 880); zBMI, z-score age-standardized body mass index; zWHR, z-score age-standardized waist-to-hip ratio. Dots/triangles and first numerical results are percentages with hypertension; ends of vertical lines and numerical results in brackets represent the 95% confidence interval of the percentages. Hypertension was defined as having either SBP ≥140 mmHg or DBP ≥90 mmHg or were on antihypertensive medication.

Values of fasting glucose (Figure 2), SBP (Figure 3) and DBP (Figure 4) were higher with increasing z-score fifths of both BMI and WHR, as were prevalences of diabetes (Figure 5) and hypertension (Figure 6). There was no evidence of a departure from a linear trend. Table 3 shows differences in means for continuous outcomes and odds ratios for binary outcomes for each increase in z-score fifths of BMI/WHR, with additional adjustment for all potential confounders. There were positive associations of both zBMI and zWHR with fasting glucose, diabetes, SBP, DBP and hypertension. These associations did not change notably with additional adjustment for smoking status, alcohol intake, wealth or physical activity level. The magnitudes of association for all outcomes were larger (nearly double for continuous outcomes) for zBMI compared with zWHR.

**Table 3.** Adjusted associations of zBMI and zWHR with glycaemic and blood pressure outcomes in the overall cohort^a^

	Difference in outcome per each increase in fifths of zBMI (95% CI)	Difference in outcome per each increase in fifths of zWHR (95% CI)
Continuous outcomes: difference in mean of outcome per fifth of zBMI/zWHR (null value = 0)		
Fasting glucose (mmol/L)		
N = 22 906	0.10 (0.09, 0.11)	0.06 (0.05, 0.07)
Systolic BP (mmHg)		
*N* = 26 682	1.47 (1.33, 1.61)	0.75 (0.61, 0.89)
Diastolic BP (mmHg)		
*N* = 26 683	1.10 (1.01, 1.19)	0.42 (0.33, 0.51)
Continuous outcomes: difference in odds ratio of outcome per fifth of zBMI/zWHR (null value=1)		
Diabetes		
*N* = 23 148 (560 with diabetes)	1.61 (1.49, 1.73)	1.46 (1.37, 1.56)
Hypertension		
*N*= 27 880 (4090 with hypertension)	1.34 (1.30, 1.37)	1.10 (1.08, 1.13)

Diabetes was defined as having any of the following: fasting glucose ≥7.0 mmol/l, currently taking antidiabetic medication or a self-report of a previous diabetes diagnosis. Hypertension was defined as having either SBP ≥140 mmHg or a DBP ≥90 mmHg or on antihypertensive medication.

CI, confidence interval; zBMI, z-score age-standardized body mass index; zWHR, z-score age-standardized waist-to-hip ratio; BP, blood pressure.

a adjusted for smoking status, alcohol intake, wealth and physical activity level.

### Associations of BMI and WHR with glycaemic and BP outcomes in gender and area of residence strata

There were positive linear associations of zBMI and zWHR with most outcomes in all four strata—rural women, rural men, urban women, urban men—but these stratified results highlighted notable differences in magnitudes of associations between rural and urban residents and between women and men (Table 4).

**Table 4.** Adjusted associations of zBMI and zWHR with glycaemic and blood pressure outcomes stratified by gender and area of residence^a^

	Difference in outcome per each increase in fifths of zBMI (95% CI)	Difference in outcome per each increase in fifths of zWHR (95% CI)
Difference in mean fasting glucose (mmol/L)		
Rural women (*N* = 6660)	0.09 (0.08, 0.11)	0.05 (0.03, 0.06)
Rural men (*N* = 4974)	0.08 (0.05, 0.10)	0.04 (0.02, 0.07)
Urban women (*N* = 7407)	0.11 (0.09, 0.13)	0.09 (0.07, 0.11)
Urban men (*N* = 3865)	0.11 (0.09, 0.14)	0.10 (0.08, 0.12)
*P*-values for difference between genders	0.5122	0.4337
*P*-values for difference between areas	0.0259	<0.001
Difference in mean systolic BP (mmHg)		
Rural women (*N* = 7144)	1.23 (0.97, 1.49)	−0.27 (-0.52,-0.01)
Rural men (*N* = 5720)	2.55 (2.21, 2.90)	1.01 (0.66, 1.35)
Urban women (*N* = 8786)	1.66 (1.42, 1.89)	1.03 (0.79, 1.28)
Urban men (*N* = 5032)	2.86 (2.53, 3.20)	1.48 (1.15, 1.81)
*P*-values for difference between genders	<0.001	<0.001
*P*-values for difference between areas	0.0904	<0.001
Difference in mean diastolic BP (mmHg)		
Rural women (*N* = 7144)	0.85 (0.68, 1.02)	0.05 (-0.12, 0.21)
Rural men (*N* = 5720)	0.89 (0.67, 1.12)	0.52 (0.29, 0.74)
Urban women (*N* = 8787)	1.24 (1.08, 1.39)	0.84 (0.68, 1.00)
Urban men (*N* = 5032)	1.39 (1.17, 1.61)	0.93 (0.72, 1.15)
*P*-values for difference between genders	0.4219	0.0013
*P*-values for difference between areas	<0.001	<0.001
Odds ratio of diabetes		
Rural women (*N* = 6711, 123 with diabetes)	1.71 (1.47, 2.00)	1.32 (1.15, 1.52)
Rural men (*N* = 5007, 84 with diabetes)	1.39 (1.18, 1.65)	1.50 (1.22, 1.85)
Urban women (*N* = 7503, 219 with diabetes)	1.90 (1.63, 2.22)	1.51 (1.38, 1.66)
Urban men (*N* = 3927, 132 with diabetes)	1.53 (1.33, 1.77)	1.94 (1.63, 2.31)
*P*-values for difference between genders	0.0117	0.0079
*P*-values for difference between areas	0.3120	0.0536
Odds ratio of hypertension		
Rural women (*N* = 7528, 1098 with diabetes)	1.25 (1.19, 1.31)	0.95 (0.90, 0.99)
Rural men (*N* = 5849, 789 with diabetes)	1.42 (1.33, 1.51)	1.17 (1.10, 1.25)
Urban women (*N* = 9290, 1343 with diabetes)	1.38 (1.31, 1.44)	1.15 (1.11, 1.20)
Urban men (*N* = 5213, 860 with diabetes)	1.47 (1.38, 1.56)	1.30 (1.23, 1.38)
*P*-values for difference between genders	0.0001	<0.001
*P*-values for difference between areas	0.0543	<0.001

Diabetes was defined as having any of the following: fasting glucose ≥7.0 mmol/l, currently taking antidiabetic medication or a self-report of a previous diabetes diagnosis. Hypertension was defined as having either SBP ≥140  mmHg or DBP ≥90 mmHg or on antihypertensive medication.

CI, confidence interval; zBMI, z-score age-standardized body mass index; zWHR, z-score age-standardized waist-to-hip ratio; BP, blood pressure.

a Adjusted for smoking status, alcohol intake, wealth and physical activity level.

For the associations with difference in mean fasting glucose and odds of diabetes, positive associations were generally stronger for zBMI than zWHR, but the differences between these two adiposity measurements were less marked in urban residents. For men, the increase in odds of diabetes for each greater fifth of adiposity was greater for zWHR than zBMI (Table 4). The magnitudes of association of both zBMI and zWHR with fasting glucose and odds of diabetes were greater in urban than rural residents. For the odds of diabetes, the magnitudes of association with zBMI were higher in women compared with men, but the associations with zWHR were higher in men.

For the differences in mean SBP and DBP and odds of hypertension, the associations were also stronger for zBMI than for zWHR (Table 4). zWHR was negatively associated with SBP in rural female residents; all other associations were positive. As with glycaemic outcomes, associations of the adiposity measures with BP were stronger in urban than rural residents. However, adiposity was more strongly associated with BP outcomes in men than in women.

For the associations with zBMI, there was statistical evidence that associations of SBP and diabetes differed by gender and that associations of fasting glucose and DBP differed by area of residence. The association between zBMI and hypertension differed by both gender and area of residence. Associations with WHR and SBP, DBP, diabetes and hypertension differed by both gender and area of residence. When participants who self-reported a previous diagnosis of hypertension but did not have elevated BPs were excluded from fully adjusted stratified analyses, there was little to no difference in the odds of hypertension (Supplementary Table 6, available as Supplementary data at *IJE* online). When fifths of zBMI and zWHR were determined within the four strata of area of residence and gender, there were only minor decreases in the difference in mean systolic BP, diastolic BP and odds ratio of diabetes among men—especially rural male residents (Supplementary Table 7, available as Supplementary data at *IJE* online). With adjustment (in addition to all other confounders) for the number of live births in women, results are not materially affected compared with those without adjustment (Supplementary Table 8, available as Supplementary data at *IJE* online).

Supplementary Tables 3 and 5 (available as Supplementary data at *IJE* online) show the results for zWC and zWHtR. For most outcomes, associations of both zWC and zWHtR were similar in magnitude to those of zBMI (i.e. they were stronger than those for zWHR). The two exceptions were that zWHtR was associated with systolic blood pressure with a magnitude that was similar to that of zWHtR and weaker than zBMI, and both zWC and zWHtR were associated with diabetes with a magnitude that was somewhat stronger than zBMI.

## Discussion

In this large study of Malawian adults, we have shown positive linear associations of BMI and WHR with adverse glycaemic and blood pressure outcomes in the overall cohort, and also within strata of female and male rural residents and female and male urban residents. In the overall cohort, and for most strata, the associations were stronger across z-score fifths for BMI than they were for WHR, suggesting that BMI might be a more useful measure of cardiometabolic risk than WHR in this population.

Magnitudes of association differed by gender and area of residence, such that for the associations of BMI and WHR with glycaemic outcomes, the associations were stronger in urban than rural residents, with little difference between women and men. The associations of zBMI and zWHR with BP-related outcomes were also stronger in urban than rural residents and we observed stronger associations in men compared with women for BP-related outcomes. These findings changed little after adjustment for smoking status, alcohol intake, physical activity and wealth, or with additional adjustment for number of live births. We did not adjust for breastfeeding as the vast majority (98%) of women in this population, as in other SSA countries, have breastfed and therefore this characteristic cannot explain the association of adiposity with cardiometabolic outcomes in these populations.

The greater positive magnitude of association of BMI and WHR with glycaemic and BP outcomes in urban residents is particularly of concern, given that increases in rates of obesity and overweight are greater in urban than rural areas. Our finding of more adverse distributions of cardiovascular risk factors in urban combined with rural residents is consistent with findings from studies in several other SSA countries.^11^^,^^13^ However, few studies have examined the associations of adiposity with risk factors as we have done here, and to our knowledge only one previous study has noted differences in association magnitudes between rural and urban residents. In the Cameroonian study, there appeared to be a positive association of BMI with high fasting glucose, insulin, triglycerides and BP in urban residents (*N *= 959), but an inverse association in rural residents (*N *= 669), whereas we saw positive linear associations in both groups but these were weaker in rural residents.^8^ That study was conducted over 20 years ago, when levels of overweight and obesity were much lower than currently in SSA. Nonetheless the possibility, from that study and ours, that greater adiposity has a stronger positive association with adverse cardiometabolic health in urban than in rural areas is concerning, as urbanization continues at pace in SSA. The already stretched health services in these countries could struggle to cope with a substantial burden of cardiometabolic illness.

Why associations are stronger in urban compared with rural populations is unclear. In our study, the rural populations are predominantly subsistence farmers and/or from fishing communities, and hence they and previous generations have high levels of physical activity and a diet that is largely fruit, vegetable and fish based. Urban residents are likely to be less active, and in urban areas there is a greater availability of processed food and lower availability of fresh fruit and vegetables. However, for these to contribute to the differences in associations that we observe (as opposed to differences in distributions) would imply some aspect of diet or physical activity interacting with adiposity in terms of its association with blood pressure and glycaemic outcomes, which has not been observed in high-income populations. The factors that might explain the greater association of adiposity with outcomes in urban compared with rural residents, which we and a previous study from Cameroon have observed, need further mechanistic studies.

A priori we considered WHR to be the most appropriate measure of central adiposity that was distinct from total adiposity as reflected in BMI. However, we also present the findings for WC and WHtR in Supplementary materials (available as Supplementary data at *IJE* online). Given the strong correlations of these measures with BMI, it is not surprising that they have similar magnitudes of associations with outcomes to those seen for BMI (and hence stronger than those seen for WHR). Distinguishing the relative contributions of BMI, WC and WHtR to cardiometabolic outcomes in SSA populations will require extremely large numbers because of problems of co-linearity between these measures.

The key strengths of this study include large sample size, inclusion of both urban and rural residents, and high quality collection of data which included fasting blood samples, which enabled us to assess associations with fasting glucose and diabetes. A key limitation is the cross-sectional nature of this study and hence the possibility that our findings are wholly, or partly, due to reverse causality and/or residual confounding. Evidence from Mendelian randomization and randomized control trials (both less prone to reverse causality or confounding) in European original participants suggests that greater adiposity increases risks of diabetes and hypertension, and it would seem reasonable to assume that is the case here.^4–6^^,^^14^^,^^15^

Despite finding higher levels of overweight and obesity in urban compared with rural residents, the distributions of physical activity, smoking and alcohol were similar in the two groups. These lifestyle factors are self-reported and the categories available are quite broad, and therefore important differences might have been missed. It is possible that misclassification resulting from self-report explains some of the differences between gender and residence strata. Furthermore, we do not have information on dietary intake, which we anticipate is different between rural and urban residents. Future large-scale studies in SSA populations which are able to measure physical activity objectively (with accelerometers) and dietary intake would be valuable.

Whereas there were very few missing data for the vast majority of measurements, 17% did not have a valid fasting blood glucose sample and therefore were excluded from analyses with glycaemic-related conditions. Those without fasting glucose differed from those with it, but the magnitudes of these differences were small, and we adjusted for these characteristics. Our results would be biased if, in those without fasting glucose, associations were markedly different from those found here in participants with fasting glucose, even after taking into account age, gender, rural/urban residence, smoking status, alcohol intake, physical activity level and wealth. Although we have no way of ruling this out, we cannot see why this would be the case.

In conclusion, our novel findings, which to our knowledge have not been previously explored in a SSA population, suggest that BMI is a useful measure for identifying those at risk of adverse cardiometabolic outcomes in SSA. Our results highlight the potential for major increases in adverse cardiometabolic health in urban areas of SSA, unless concerted efforts for preventing increases in adiposity are successfully introduced. Although this might also apply to other low-income countries in the SSA region, there is also evidence of increasing risk in rural communities in higher-income countries such as South Africa,^16–18^ and so any interventions should not disadvantage rural residents.

## Supplementary data

Supplementary data are available at *IJE* online.

## Funding

This work was supported by the Wellcome Trust (WT098610) who funded the study and had no role in the study design, data collection and analysis, decision to publish, or preparation of the manuscript (098610/Z/12/Z). D.A.L. is supported by the Medical Research Council (MC_UU_12013/5) and by the National Institute for Health Research (NF-SI-0611–10196). L.S. is supported by a Wellcome Senior Research Fellowship in Clinical Science (098504/Z/12/Z).

## Supplementary Material

Supplementary Data
