# Supplementary material for: How does the association of general and central adiposity with glycaemia and blood pressure differ by gender and area of residence in a Malawian population: a cross-sectional study
Source: Int J Epidemiol. 2018 Apr 10;47(3):887–98. doi: 10.1093/ije/dyy047 (PMC6005143; doi:10.1093/ije/dyy047)
Supplement: Supplementary Data [file dyy047_supp.zip › dyy047-suppl_data/ije-2017-06-0688-File012.docx]

Supplementary Materials

| **Contents** | **Page #** | **Description** |
| --- | --- | --- |
| **SText** |  |  |
| Standard protocols | 2 | Standardised protocols used by trained field workers and phlebotomy nurses to collect all questionnaires and measurements |
| Categorisation of potential confounders | 3 | Details on how potential confounders were categorised for analyses |
| **STables** |  |  |
| STable 1 | 5 | Relationship of BMI, WHR, gender, age, area of residence, wealth, smoking status, alcohol intake, and physical activity level with missing glucose values |
| STable 2 | 7 | Unadjusted associations of zBMI and zWHR with glycaemic and blood pressure outcomes in the overall cohort |
| STable 3 | 8 | Unadjusted and adjusted associations of zWC and zWHtR with glycaemic and blood pressure outcomes in the overall cohort |
| STable 4 | 9 | Unadjusted associations of zBMI and zWHR with glycaemic and blood pressure outcomes, stratified by gender and area of residence |
| STable 5 | 10 | Unadjusted and adjusted associations of zWC and zWHtR with glycaemic and blood pressure outcomes stratified by gender and area of residence |
| STable 6 | 12 | Adjusted associations of zBMI, zWHR, zWC, and zWHtR with hypertension after removing participants who self-reported a previous hypertension diagnosis but did not have elevated BP in our assessments |
| STable 7 | 13 | Adjusted associations of zBMI, zWHR, zWC, and zWHtR (divided into fifths determined within our area of residence and gender strata) with glycaemic and blood pressure outcomes stratified by gender and area of residence |
| STable 8 | 14 | Main adjusted associations of zBMI, zWHR, zWC, and zWHtR with glycaemic and blood pressure outcomes among rural and urban women and additionally adjusted for number of live births |
| **SFigures** |  |  |
| SFig. 1 | 17 | Mean fasting glucose per fifth of zWC and zWHtR (N=22906) |
| SFig. 2 | 17 | Mean systolic blood pressure per fifth of zWC and zWHtR (N=26682) |
| SFig. 3 | 17 | Mean diastolic blood pressure per fifth of zWC and zWHtR (N=26683) |
| SFig. 4 | 17 | Percent with diabetes per fifth of zWC and zWHtR (N=23148) |
| SFig. 5 | 17 | Percent with hypertension per fifth of zWC and zWHtR (N=27880) |

# SText

## Standard protocols

Anthropometric data (weight, height, waist, hip and mid-upper arm circumference) were measured in duplicate, with participants in light clothing and unshod. Weight was measured on portable scales which were calibrated with a standard 1kg weight at the start of each data collection session; height was measured with a portable stadiometer with the participant’s head in the correct position. All circumferences were measured with flexible tables of appropriate size. The waist circumference was taken as the mid-point between the lower rib and pelvic crest, and hip-circumference as the largest circumference below the pelvic crest. Mid-upper arm circumference (MUAC) was measured at the midpoint between the top of the shoulder and the tip of the elbow. The mean of the two measurements was used in all analyses.

SBP and DBP were measured after 30-minutes rest with the participant seated and their arm supported; the appropriate cuff-size based on arm circumference was used. Measurements were taken in triplicate with 5-minutes resting time in between each, using a portable electronic device (OMRON Healthcare Co., LTd. HEB-7211-E, Model M6). The average of the last two readings was used in all analyses to allow for the phenomenon of raised BP during an unfamiliar procedure affecting the first measure. In interviews, participants were asked whether they had ever previously been diagnosed with high blood pressure and about any regular medication (used medicine bottles were inspected where possible).

Participants consenting to blood draws were given a morning appointment and asked to fast overnight (consuming nothing but water until after the sample was taken). Venous blood samples were taken and processed in MEIRU laboratories at each site within four-hours of sampling. Plasma fasting glucose was measured using automated analysers (Beckman Coulter Chemistry analyser, Model: AU480). The laboratories at both sites participate in an external quality control scheme (Thistle, RSA) and internal controls were conducted, with exchange of samples between the two sites for repeat testing to ensure consistency of measurements. Participants were asked in interviews whether they had previously been diagnosed with diabetes and about use of any regular medication.

## Categorisation of potential confounders

Smoking was categorized as never, previous, current light smoker (<6 cigs/day), or heavy smoker (≥6 cigs/day); those who reported stopping within the last 6 months were considered current smokers at the level they reported before stopping. Alcohol intake was categorized as none in the last year, light or irregular (<8 units/wk), moderate (8-21 units/wk), or heavy (>21 units/wk). Given the majority of participants reported never smoking, with small numbers in all other categories, with similar imbalance of alcohol consumption (see Table 1), in multivariable regression analyses these two were used as binary variables: never smoking – yes versus no; and no alcohol in the last year – yes versus no. Based on WHO cut-offs of METabolic Equivalent scores, physical activity was categorized as light (<4 METs), moderate (4-7 METs), or heavy (>7METs) per day. The number of live births was categorised as zero, one, two, three, and four or more.

We a priori chose wealth index as our measure of SEP, as we feel this is will strongly reflect, in this population, access to different dietary and other lifestyle choices that will influence adiposity and outcomes. The wealth index was derived by combining responses to a series of yes/no questions (see below) on commonly owned household assets and ascribing monetary value to those assets, creating a cumulative continuous variable with low values implying poverty and high values implying one has multiple valuable assets. The score was then divided into fifths where the lowest fifth was assets of value 0-97,000 Malawian Kwacha (MWK), or £0-102, and the top fifth was assets of value greater than 553,500 MWK, or £558.

Questions:

1. Does the participant own at least one paraffin lamp?
2. Does the participant own at least one television?
3. Does the participant own at least one mobile phone?
4. Does the participant own at least one telephone landline?
5. Does the participant own at least one bed with a mattress?
6. Does the participant own at least one sofa set?
7. Does the participant own at least one refrigerator?
8. Does the participant own at least one table and at least one chair?
9. Does the participant own at least one electric or gas cooking stove?
10. Does the participant own at least one motorcycle?
11. Does the participant own at least one car/private vehicle?
12. Does the participant own at least one bicycle?
13. Does the participant own at least one cow?
14. Does the participant own at least one oxcart?

# STables

STable 1: Relationship of BMI, WHR, gender, age, area of residence, wealth, smoking status, alcohol intake, and physical activity level with missing glucose values

|  |  | **Total N=4726 (%)** | **Odds of not having a fasting glucose measure (95% CI)** |
| --- | --- | --- | --- |
| **Gender** | Women | 2600 (15.5) | ref |
| N(%) | Men | 2126 (19.2) | 1.30 (1.22,1.39) |
| **Residence** | Rural | 1667 (12.5) | ref |
| N(%) | Urban | 3059 (21.1) | 1.88 (1.76,2.00) |
| **Age** | mean (±SD) | 35.1 (16.0) | 1.00 (0.99,1.00) |
| **Wealth fifths** | 1 (<£102) | 915 (18.3) | ref |
| N(%) | 2 (£102-£158) | 933 (15.6) | 0.82 (0.74,0.91) |
|  | 3 (£158-£307) | 916 (17.1) | 0.92 (0.83,1.02) |
|  | 4 (£307-£583) | 1053 (16.3) | 0.87 (0.78,0.95) |
|  | 5 (>£583) | 805 (16.6) | 0.89 (0.80,0.98) |
|  | missing | 104 (45.4) | 3.70 (2.83,4.85) |
| **Smoking status** | Never | 4293 (16.6) | ref |
| N(%) | Ex-smoker | 170 (23.9) | 1.58 (1.32,1.88) |
|  | Light (<6 cigs/day) | 161 (19.9) | 1.25 (1.05,1.49) |
|  | Heavy (≥6 cigs/day) | 102 (20.9) | 1.33 (1.07,1.66) |
| **Alcohol intake** | None in last year | 3692 (16.3) | ref |
| N(%) | Light (<8 units/wk) | 745 (18.6) | 1.17 (1.07,1.27) |
|  | Moderate (8-21 units/wk) | 223 (22.5) | 1.49 (1.28,1.74) |
|  | Heavy (>21 units/wk) | 66 (26.6) | 1.86 (1.40,2.47) |
| **Physical activity level** | Low (<4 METs/day) | 252 (24.1) | ref |
| N(%) | Moderate (4-7 METs/day) | 571 (20.2) | 0.79 (0.67,0.94) |
|  | High (>7 METs/day) | 3903 (16.3) | 0.61 (0.53,0.71) |
| **Systolic BP** | mean (±SD) | 123.4 (17.6) | 1.00 (1.00,1.00) |
| **Diastolic BP** | mean (±SD) | 73.2 (10.8) | 1.00 (0.99,1.00) |
| **BMI** | median (IQR) | 22.0 (20.2,24.8) | 0.97 (0.96,0.98) |
| N(%) | 1 (lowest) | 1046 (18.8) | ref |
|  | 2 | 996 (17.9) | 0.94 (0.85,1.04) |
|  | 3 | 926 (16.6) | 0.86 (0.78,0.95) |
|  | 4 | 905 (16.3) | 0.84 (0.76,0.92) |
|  | 5 (highest) | 838 (15.1) | 0.77 (0.69,0.85) |
|  | missing | 15 (26.8) | 1.58 (0.87,2.87) |
| **WHR** | median (IQR) | 0.83 (0.79,0.87) | 0.27 (0.17,0.43) |
| N(%) | 1 (lowest) | 1027 (18.4) | ref |
|  | 2 | 963 (17.3) | 0.93 (0.84,1.02) |
|  | 3 | 924 (16.6) | 0.88 (0.80,0.97) |
|  | 4 | 898 (16.1) | 0.85 (0.77,0.94) |
|  | 5 (highest) | 904 (16.2) | 0.86 (0.78,0.95) |
|  | missing | 10 (23.3) | 1.34 (0.66,2.73) |

OR: odds ratio; CI: confidence interval; SD: standard deviation; IQR: interquartile range (25%, 75%); BMI: body mass index; WHR: waist to-hip ratio; BP: blood pressure; ^a^row percentages: those that are missing out of total in each category.

STable 2: Unadjusted associations of zBMI and zWHR with glycaemic and blood pressure outcomes in the overall cohort

|  | **Difference in outcome per each increase in fifths of zBMI (95% CI)** | **Difference in outcome per each increase in fifths of zWHR (95% CI)** |
| --- | --- | --- |
| **Continuous outcomes - difference in mean of outcome per fifth of zBMI/zWHR (null value=0)** | | |
| **Fasting glucose (mmol/L)** |  |  |
| N=22906 | 0.10 (0.09,0.11) | 0.05 (0.04,0.06) |
| **Systolic BP (mmHg)** |  |  |
| N=26682 | 1.33 (1.20,1.47) | 0.84 (0.70,0.98) |
| **Diastolic BP (mmHg)** |  |  |
| N=26683 | 1.05 (0.96,1.13) | 0.44 (0.35,0.53) |
| **Continuous outcomes - difference in odds ratio of outcome per fifth of zBMI/zWHR (null value=1)** | | |
| **Diabetes** |  |  |
| N=23148 (560 with diabetes) | 1.70 (1.59,1.83) | 1.44 (1.35,1.54) |
| **Hypertension** |  |  |
| N=27880 (4090 with hypertension) | 1.34 (1.30,1.37) | 1.11 (1.08,1.13) |

CI: confidence interval; zBMI: z-score; age-standardised body mass index; zWHR: z-score, age-standardised waist-to-hip ratio; BP: blood pressure. Diabetes was defined as having any of the following: fasting glucose ≥7.0 mmol/L currently taking antidiabetic medication, or a self-report of a previous diabetes diagnosis. Hypertension was defined as having either a SBP ≥140mmHg or a DBP ≥90mmHg or were on antihypertensive medication.

STable 3: Unadjusted and adjusted associations of zWC and zWHtR with glycaemic and blood pressure outcomes in the overall cohort

|  | **Difference in outcome per each increase in fifths of zWC(95%CI)** | | **Difference in outcome per each increase in fifths of zWHtR (95%CI)** | |
| --- | --- | --- | --- | --- |
| **Outcome** | **Unadjusted** | **Fully adjusted*** | **Unadjusted** | **Fully adjusted*** |
| **Continuous outcomes - difference in mean outcome per fifth of zWC/zWHtR (null value=0)** | | | | |
| **Fasting glucose (mmol/L)** |  |  |  |  |
| N=22906 | 0.11 (0.10,0.12) | 0.11 (0.09,0.11) | 0.09 (0.08,0.10) | 0.09 (0.08,0.10) |
| **Systolic BP (mmHg)** |  |  |  |  |
| N=26682 | 1.49 (1.35,1.62) | 1.48 (1.34,1.61) | 0.60 (0.46,0.73) | 0.81 (0.67,0.95) |
| **Diastolic BP (mmHg)** |  |  |  |  |
| N=26683 | 1.10 (1.01,1.18) | 1.09 (1.00,1.17) | 0.97 (0.88,1.05) | 1.07 (0.98,1.15) |
| **Binary outcomes - odds ratio of outcome per fifth of zWC/zWHtR (null value=1)** | | | | |
| **Diabetes** |  |  |  |  |
| N=23148 (560 with diabetes) | 1.89 (1.76,2.04) | 1.79 (1.66,1.93) | 1.74 (1.62,1.87) | 1.72 (1.59,1.85) |
| **Hypertension** |  |  |  |  |
| N=27880 (4090 with hypertension) | 1.33 (1.30,1.36) | 1.31 (1.28,1.35) | 1.29 (1.25,1.32) | 1.30 (1.27,1.33) |

CI: confidence interval; zWC: z-score; age-standardised waist-circumference; zWHtR: z-score, age-standardised waist-to-height; BP: blood pressure; *adjusted for gender, area of residence, smoking status, alcohol intake, wealth, and physical activity level. Diabetes was defined as having any of the following: fasting glucose ≥7.0 mmol/L currently taking antidiabetic medication, or a self-report of a previous diabetes diagnosis. Hypertension was defined as having either a SBP ≥140mmHg or a DBP ≥90mmHg or were on antihypertensive medication.

STable 4: Unadjusted associations of zBMI and zWHR with glycaemic and blood pressure outcomes in the overall cohort, stratified by gender and area of residence

|  | **Difference in outcome per each increase in fifths of zBMI (95% CI)** | **Difference in outcome per each increase in fifths of zWHR (95% CI)** |
| --- | --- | --- |
| **Difference in mean fasting glucose (mmol/L)** |  |  |
| Rural women (N=6660) | 0.10 (0.08,0.11) | 04.04 (0.03,0.06) |
| Rural men (N=4974) | 0.08 (0.06,0.10) | 0.05 (0.02,0.07) |
| Urban women (N=7407) | 0.12 (0.10,0.13) | 0.08 (0.07,0.10) |
| Urban men (N=3865) | 0.13 (0.10,0.15) | 0.11 (0.09,0.13) |
| P-values for difference between gender | 0.8847 | 0.157 |
| P-values for difference between area | 0.015 | <0.001 |
| **Difference in mean systolic BP (mmHg)** |  |  |
| Rural women (N=7144) | 1.22 (0.96,1.48) | -0.29 (-0.55,0.04) |
| Rural men (N=5720) | 2.50 (2.15,2.84) | 1.05 (0.70,1.39) |
| Urban women (N=8786) | 1.66 (1.43,1.89) | 1.03 (0.79,1.28) |
| Urban men (N=5032) | 2.98 (2.65,3.31) | 1.58 (1.25,1.91) |
| P-values for difference between gender | <0.001 | <0.001 |
| P-values for difference between area | 0.0567 | <0.001 |
| **Difference in mean diastolic BP (mmHg)** |  |  |
| Rural women (N=7144) | 0.86 (0.69,1.03) | 0.03 (-0.14,0.19) |
| Rural men (N=5720) | 0.84 (0.62,1.07) | 0.53 (0.31,0.76) |
| Urban women (N=8787) | 1.26 (1.11,1.41) | 0.82 (0.67,0.98) |
| Urban men (N=5032) | 1.47 (1.26,1.69) | 1.03 (0.82,1.25) |
| P-values for difference between gender | 0.3452 | 0.0001 |
| P-values for difference between area | <0.001 | <0.001 |
| **Odds ratio of diabetes** |  |  |
| Rural women (N=6711, 123 with diabetes) | 1.79 (1.53,2.10) | 1.29 (1.13,1.48) |
| Rural men (N=5007, 84 with diabetes) | 1.46 (1.23,1.73) | 1.55 (1.26,1.91) |
| Urban women (N=7503, 219 with diabetes) | 2.06 (1.76,2.42) | 1.51 (1.38,1.66) |
| Urban men (N=3927, 132 with diabetes) | 1.71 (1.48,1.98) | 2.14 (1.79,2.55) |
| P-values for difference between gender | 0.0424 | 0.0003 |
| P-values for difference between area | 0.2399 | 0.0115 |
| **Odds ratio of hypertension** |  |  |
| Rural women (N=7528, 1098 with diabetes) | 1.26 (1.21,1.33) | 0.94 (0.89,0.98) |
| Rural men (N=5849, 789 with diabetes) | 1.41 (1.33,1.50) | 1.18 (1.11,1.26) |
| Urban women (N=9290, 1343 with diabetes) | 1.40 (1.34,1.47) | 1.15 (1.10,1.20) |
| Urban men (N=5213, 860 with diabetes) | 1.54 (1.45,1.63) | 1.35 (1.27,1.43) |
| P-values for difference between gender | <0.001 | <0.001 |
| P-values for difference between area | 0.0243 | <0.001 |

CI: confidence interval; zBMI: z-score; age-standardised body mass index; zWHR: z-score, age-standardised waist-to-hip ratio; BP: blood pressure. Diabetes was defined as having any of the following: fasting glucose ≥7.0 mmol/L currently taking antidiabetic medication, or a self-report of a previous diabetes diagnosis. Hypertension was defined as having either a SBP ≥140mmHg or a DBP ≥90mmHg or were on antihypertensive medication.

STable 5: Unadjusted and adjusted associations of zWC and zWHtR with glycaemic and blood pressure outcomes stratified by gender and area of residence

|  | **Difference in outcome per each increase in fifths of zWC (95%CI)** | | **Difference in outcome per each increase in fifths of zWHtR (95%CI)** | |
| --- | --- | --- | --- | --- |
|  | **Unadjusted** | **Fully adjusted*** | **Unadjusted** | **Fully adjusted*** |
| **Difference in mean fasting glucose (mmol/L)** | | |  |  |
| Rural women (N=6660) | 0.09 (0.07,0.10) | 0.09 (0.07,0.10) | 0.08 (0.07,0.10) | 0.08 (0.07,0.10) |
| Rural men (N=4974) | 0.08 (0.06,0.10) | 0.08 (0.05,0.10) | 0.09 (0.07,0.11) | 0.09 (0.06,0.11) |
| Urban women (N=7407) | 0.12 (0.10,0.13) | 0.12 (0.10,0.13) | 0.12 (0.10,0.13) | 0.11 (0.10,0.13) |
| Urban men (N=3865) | 0.13 (0.10,0.15) | 0.12 (0.09,0.14) | 0.13 (0.10,.16) | 0.12 (0.09,0.14) |
| P-values for difference between gender | 0.7863 | 0.4348 | 0.5478 | 0.9534 |
| P-values for difference between area | <0.001 | 0.0001 | 0.0002 | 0.0010 |
| **Difference in mean systolic BP (mmHg)** | | |  |  |
| Rural women (N=7144) | 0.83 (0.58,1.08) | 0.72 (0.46,0.97) | 0.69 (0.42,0.98) | 0.40 (0.13,0.68) |
| Rural men (N=5720) | 2.39 (2.07,2.71) | 2.44 (2.11,2.77) | 2.19 (1.84,2.53) | 2.24 (1.89,2.60) |
| Urban women (N=8786) | 1.66 (1.45,1.88) | 1.64 (1.42,1.85) | 1.62 (1.39,1.85) | 1.57 (1.34,1.80) |
| Urban men (N=5032) | 2.62 (2.30,2.95) | 2.53 (2.20,2.85) | 2.77 (2.43,3.11) | 2.64 (2.30,2.99) |
| P-values for difference between gender | <0.001 | <0.001 | <0.001 | <0.001 |
| P-values for difference between area | 0.0001 | 0.0001 | <0.001 | <0.001 |
| **Difference in mean diastolic BP (mmHg)** | | |  |  |
| Rural women (N=7144) | 0.66 (0.50,0.83) | 0.64 (0.47,0.80) | 0.55 (0.38,0.73) | 0.47 (0.30,0.65) |
| Rural men (N=5720) | 0.96 (0.75,1.17) | 0.97 (0.76,1.19) | 1.10 (0.87,1.33) | 1.12 (0.89,1.36) |
| Urban women (N=8787) | 1.33 (1.19,1.47) | 1.30 (1.16,1.45) | 1.31 (1.16,1.46) | 1.28 (1.13,1.43) |
| Urban men (N=5032) | 1.49 (1.28,1.70) | 1.40 (1.18,1.61) | 1.81 (1.59,2.03) | 1.74 (1.52,1.96) |
| P-values for difference between gender | 0.0756 | 0.1132 | <0.001 | <0.001 |
| P-values for difference between area | <0.001 | <0.001 | <0.001 | <0.001 |
| **Odds ratio of diabetes** | |  |  |  |
| Rural women (N=6711, 123 with diabetes) | 1.69 (1.47,1.93) | 1.74 (1.48,2.04) | 1.70 (1.49,1.95) | 1.84 (1.52,2.23) |
| Rural men (N=5007, 84 with diabetes) | 1.45 (1.24,1.70) | 1.44 (1.21,1.71) | 1.46 (1.26,1.69) | 1.49 (1.26,1.77) |
| Urban women (N=7503, 219 with diabetes) | 2.08 (1.82,2.36) | 2.05 (1.78,2.36) | 2.09 (1.83,2.39) | 2.15 (1.84,2.52) |
| Urban men (N=3927, 132 with diabetes) | 1.94 (1.67,2.26) | 1.78 (1.52,2.08) | 1.80 (1.58,2.06) | 1.68 (1.46,1.93) |
| P-values for difference between gender | 0.1161 | 0.0278 | 0.0298 | 0.003 |
| P-values for difference between area | 0.0009 | 0.0134 | 0.0092 | 0.0634 |
| **Odds ratio of hypertension** | | |  |  |
| Rural women (N=7528, 1098 with hypertension) | 1.17 (1.12,1.23) | 1.14 (1.09,1.19) | 1.17 (1.12,1.23) | 1.11 (1.06,1.17) |
| Rural men (N=5849, 789 with hypertension) | 1.40 (1.32,1.49) | 1.39 (1.31,1.48) | 1.43 (1.35,1.52) | 1.43 (1.34,1.53) |
| Urban women (N=9290, 1343 with hypertension) | 1.38 (1.32,1.43) | 1.35 (1.29,1.40) | 1.39 (1.33,1.45) | 1.35 (1.29,1.42) |
| Urban men (N=5213, 860 with hypertension) | 1.53 (1.44,1.62) | 1.47 (1.39,1.56) | 1.54 (1.46,1.63) | 1.47 (1.39,1.56) |
| P-values for difference between gender | <0.001 | <0.001 | <0.001 | <0.001 |
| P-values for difference between area | <0.001 | <0.001 | 0.0005 | 0.0001 |

CI: confidence interval; zWC: z-score; age-standardised waist-circumference; zWHtR: z-score, age-standardised waist-to-height ratio; BP: blood pressure; *adjusted for smoking status, alcohol intake, wealth, and physical activity level. Diabetes was defined as having any of the following: fasting glucose ≥7.0 mmol/L currently taking antidiabetic medication, or a self-report of a previous diabetes diagnosis. Hypertension was defined as having either a SBP ≥140mmHg or a DBP ≥90mmHg or were on antihypertensive medication.

STable 6: Adjusted associations of zBMI, zWHR, zWC, and zWHtR with hypertension after removing participants who self-reported a previous hypertension diagnosis but did not have elevated BP in our assessments, stratified by gender and area of residence*

|  | **Difference in outcome per each increase in fifths of zBMI (95%CI)** | **Difference in outcome per each increase in fifths of zWHR (95%CI)** | **Difference in outcome per each increase in fifths of zWC (95%CI)** | **Difference in outcome per each increase in fifths of zWHtR (95%CI)** |
| --- | --- | --- | --- | --- |
| **Odds ratio of hypertension** |  |  |  |  |
| Rural women (N=7528, 1098 with hypertension) | 1.26 (1.20,1.32) | 0.95 (0.90,0.99) | 1.15 (1.09,1.20) | 1.12 (1.06,1.18) |
| Rural men (N=5849, 789 with hypertension) | 1.43 (1.34,1.52) | 1.17 (1.10,1.25) | 1.40 (1.32,1.49) | 1.44 (1.35,1.54) |
| Urban women (N=9290, 1343 with hypertension) | 1.40 (1.33,1.47) | 1.16 (1.12,1.21) | 1.37 (1.31,1.43) | 1.38 (1.31,1.44) |
| Urban men (N=5213, 860 with hypertension) | 1.48 (1.39,1.57) | 1.30 (1.23,1.38) | 1.48 (1.39,1.57) | 1.48 (1.40,1.57) |
| P-values for difference between gender | 0.0004 | <0.001 | <0.001 | <0.001 |
| P-values for difference between area | 0.0217 | <0.001 | <0.001 | <0.001 |

CI: confidence interval; zBMI: z-score, age-standardised body mass index; zWHR: z-score, age-standardised waist-to-hip ratio; zWC: z-score, age-standardised waist-circumference; zWHtR: z-score, age-standardised waist-to-height ratio; *adjusted for smoking status, alcohol intake, wealth, and physical activity level. Diabetes was defined as having any of the following: fasting glucose ≥7.0 mmol/L currently taking antidiabetic medication, or a self-report of a previous diabetes diagnosis. Hypertension was defined as having either a SBP ≥140mmHg or a DBP ≥90mmHg or were on antihypertensive medication.

STable 7: Adjusted associations of zBMI, zWHR, zWC, and zWHtR (divided into fifths determined within our area of residence and gender strata) with glycaemic and blood pressure outcomes, stratified by gender and area of residence*

|  | **Difference in outcome per each increase in fifths of zBMI (95%CI)** | **Difference in outcome per each increase in fifths of zWHR (95%CI)** | **Difference in outcome per each increase in fifths of zWC (95%CI)** | **Difference in outcome per each increase in fifths of zWHtR (95%CI)** |
| --- | --- | --- | --- | --- |
| **Difference in mean fasting glucose (mmol/L)** | | |  |  |
| Rural women (N=6660) | 0.09 (0.08,0.11) | 0.05 (0.03,0.07) | 0.09 (0.07,0.10) | 0.08 (0.06,0.10) |
| Rural men (N=4974) | 0.06 (0.04,0.08) | 0.04 (0.02,0.06) | 0.06 (0.04,0.08) | 0.06 (0.04,0.08) |
| Urban women (N=7407) | 0.12 (0.10,0.13) | 0.08 (0.06,0.09) | 0.12 (0.11,0.14) | 0.12 (0.11,0.14) |
| Urban men (N=3865) | 0.10 (0.07,0.12) | 0.10 (0.07,0.12) | 0.11 (0.09,0.13) | 0.10 (0.07,0.12) |
| P-values for difference between gender | 0.0017 | 0.8474 | 0.0071 | 0.0075 |
| P-values for difference between area | 0.0005 | <0.001 | <0.001 | <0.001 |
| **Difference in mean systolic BP (mmHg)** | | |  |  |
| Rural women (N=7144) | 1.21 (0.95,1.47) | ,0.21 (,0.47,0.05) | 0.71 (0.45,0.97) | 0.52 (0.26,0.77) |
| Rural men (N=5720) | 2.01 (1.72,2.30) | 0.85 (0.57,1.14) | 1.99 (1.70,2.28) | 1.72 (1.43,2.01) |
| Urban women (N=8786) | 1.59 (1.36,1.83) | 1.03 (0.79,1.26) | 1.70 (1.47,1.93) | 1.57 (1.33,1.80) |
| Urban men (N=5032) | 2.61 (2.30,2.92) | 1.40 (1.08,1.71) | 1.36 (1.05,1.67) | 1.24 (1.93,2.55) |
| P-values for difference between gender | <0.001 | <0.001 | <0.001 | <0.001 |
| P-values for difference between area | 0.0037 | <0.001 | <0.001 | <0.001 |
| **Difference in mean diastolic BP (mmHg)** | | |  |  |
| Rural women (N=7144) | 0.84 (0.67,1.00) | 0.08 (,0.09,0.25) | 0.66 (0.50,0.83) | 0.55 (0.38,0.71) |
| Rural men (N=5720) | 0.61 (0.43,0.80) | 0.44 (0.27,0.64) | 0.71 (0.52,0.90) | 0.77 (0.58,0.96) |
| Urban women (N=8787) | 1.29 (1.14,1.44) | 0.82 (0.67,0.97) | 1.41 (1.25,1.56) | 1.33 (1.18,1.48) |
| Urban men (N=5032) | 1.20 (0.99,1.40) | 0.91 (0.71,1.11) | 1.27 (1.07,1.48) | 1.37 (1.16,1.57) |
| P-values for difference between gender | 0.0270 | 0.0445 | 0.2789 | 0.4546 |
| P-values for difference between area | <0.001 | <0.001 | <0.001 | <0.001 |
| **Odds ratio of diabetes** | |  |  |  |
| Rural women (N=6711, 123 with diabetes) | 1.71 (1.47,2.00) | 1.36 (1.19,1.56) | 1.71 (1.47,1.99) | 1.63 (1.41,1.90) |
| Rural men (N=5007, 84 with diabetes) | 1.30 (1.11,1.52) | 1.39 (1.18,1.64) | 1.31 (1.11,1.54) | 1.35 (1.15,1.59) |
| Urban women (N=7503, 220 with diabetes) | 1.68 (1.49,1.88) | 1.55 (1.40,1.73) | 1.99 (1.75,2.27) | 2.02 (1.77,2.29) |
| Urban men (N=3927, 133 with diabetes) | 1.49 (1.29,1.72) | 1.90 (1.61,2.23) | 1.75 (1.50,2.04) | 1.70 (1.46,1.97) |
| P-values for difference between gender | 0.0107 | 0.1301 | 0.0089 | 0.0227 |
| P-values for difference between area | 0.4240 | 0.0075 | 0.0035 | 0.0027 |
| **Odds ratio of hypertension** | |  |  |  |
| Rural women (N=7528, 1098 with hypertension) | 1.24 (1.19,1.30) | 0.95 (0.91,0.99) | 1.14 (1.09,1.20) | 1.13 (1.08,1.18) |
| Rural men (N=5849, 789 with hypertension) | 1.31 (1.24,1.39) | 1.14 (1.08,1.21) | 1.30 (1.23,1.38) | 1.30 (1.22,1.37) |
| Urban women (N=9290, 1343 with hypertension) | 1.35 (1.29,1.41) | 1.16 (1.11,1.21) | 1.37 (1.31,1.44) | 1.34 (1.29,1.40) |
| Urban men (N=5213, 860 with hypertension) | 1.42 (1.34,1.50) | 1.29 (1.22,1.37) | 1.44 (1.36,1.52) | 1.42 (1.34,1.50) |
| P-values for difference between gender | 0.0689 | <0.001 | 0.0018 | 0.0006 |
| P-values for difference between area | 0.0025 | <0.001 | <0.001 | <0.001 |

CI: confidence interval; zBMI: z-score, age-standardised body mass index; zWHR: z-score, age-standardised waist-to-hip ratio; zWC: z-score, age-standardised waist-circumference; zWHtR: z-score, age-standardised waist-to-height ratio; *adjusted for smoking status, alcohol intake, wealth, and physical activity level. Diabetes was defined as having any of the following: fasting glucose ≥7.0 mmol/L currently taking antidiabetic medication, or a self-report of a previous diabetes diagnosis. Hypertension was defined as having either a SBP ≥140mmHg or a DBP ≥90mmHg or were on antihypertensive medication.

STable 8: Main adjusted associations of zBMI, zWHR, zWC, and zWHtR with glycaemic and blood pressure outcomes among rural and urban women and additionally adjusted for number of live births

|  | **Difference in outcome per each increase in fifths of zBMI (95%CI)** | | **Difference in outcome per each increase in fifths of zWHR (95%CI)** | | **Difference in outcome per each increase in fifths of zWC (95%CI)** | | **Difference in outcome per each increase in fifths of zWHtR (95%CI)** | |
| --- | --- | --- | --- | --- | --- | --- | --- | --- |
|  | **Main analyses*** | **Adjusted for number of live births** | **Main analyses*** | **Adjusted for number of live births** | **Main analyses*** | **Adjusted for number of live births** | **Main analyses*** | **Adjusted for number of live births** |
| **Difference in mean fasting glucose (mmol/L)** | | |  |  |  |  |  |  |
| Rural women (N=6170) | 0.09 (0.07,0.11) | 0.09 (0.08,0.11) | 0.05 (0.03,0.06) | 0.05 (0.03,0.06) | 0.09 (0.07,0.10) | 0.09 (0.07,0.11) | 0.08 (0.06,0.10) | 0.09 (0.07,0.11) |
| Urban women (N=6020) | 0.11 (0.09,0.13) | 0.10 (0.09,0.12) | 0.09 (0.07,0.10) | 0.08 (0.07,0.10) | 0.12 (0.10,0.13) | 0.11 (0.10, 0.13) | 0.11 (0.10,0.13) | 0.11 (0.09,0.12) |
| P-value for difference between area | 0.1463 | 0.4709 | 0.0005 | 0.0038 | 0.0076 | 0.0789 | 0.0108 | 0.1364 |
| **Difference in mean systolic BP (mmHg)** | | |  |  |  |  |  |  |
| Rural women (N=6570) | 1.25 (0.98,1.52) | 1.37 (1.11,1.64) | -0.24 (-0.51,0.02) | -0.13 (-0.39,0.13) | 0.74 (0.48,1.01) | 0.95 (0.69,1.21) | 0.42 (0.13,0.71) | 0.72 (0.44,1.00) |
| Urban women (N=6579) | 1.66 (1.41,1.91) | 1.52 (1.28,1.76) | 1.03 (0.78,1.29) | 0.95 (0.70,1.20) | 1.63 (1.41,1.86) | 1.51 (1.29,1.74) | 1.58 (1.34,1.82) | 1.47 (1.23,1.71) |
| P-value for difference between area | 0.0282 | 0.4171 | <0.001 | <0.001 | <0.001 | 0.0014 | <0.001 | 0.0001 |
| **Difference in mean diastolic BP (mmHg)** | | |  |  |  |  |  |  |
| Rural women (N=6570) | 0.86 (0.69,1.03) | 0.95 (0.79,1.11) | 0.05 (-0.11,0.22) | 0.11 (-0.05,0.27) | 0.65 (0.48,0.81) | 0.79 (0.63,0.95) | 0.48 (0.30,0.66) | 0.68 (0.51,0.85) |
| Urban women (N=6579) | 1.25 (1.10,1.40) | 1.10 (0.95,1.25) | 0.84 (0.68,0.99) | 0.72 (0.57,0.87) | 1.31 (1.17,1.45) | 1.16 (1.03,1.30) | 1.29 (1.14,1.44) | 1.15 (1.00,1.30) |
| P-value for difference between area | 0.0007 | 0.1705 | <0.001 | <0.001 | <0.001 | 0.0004 | <0.001 | <0.001 |
| **Odds ratio of diabetes** |  |  |  |  |  |  |  |  |
| Rural women (N=6221, 119 with diabetes) | 1.71 (1.47,2.00) | 1.73 (1.48,2.02) | 1.33 (1.16,1.53) | 1.33 (1.16,1.53) | 1.74 (1.49,2.05) | 1.77 (1.51,2.08) | 1.85 (1.52,2.24) | 1.89 (1.56,2.29) |
| Urban women (N=7018, 221 with diabetes) | 1.89 (1.62,2.22) | 1.79 (1.52,2.09) | 1.51 (1.38,1.66) | 1.46 (1.32,1.60) | 2.05 (1.78,2.35) | 1.94 (1.68,2.24) | 2.15 (1.84,2.52) | 2.02 (1.72,2.36) |
| P-value for difference between area | 0.3741 | 0.7563 | 0.1350 | 0.3111 | 0.1421 | 0.3908 | 0.2333 | 0.6190 |
| **Odds ratio of hypertension** | | |  |  |  |  |  |  |
| Rural women (N=6944, 1064 with hypertension) | 1.26 (1.20,1.32) | 1.28 (1.22,1.35) | 0.95 (0.91,0.99) | 0.95 (0.91,1.00) | 1.14 (1.09,1.20) | 1.18 (1.12,1.23) | 1.12 (1.06,1.18) | 1.16 (1.10,1.22) |
| Urban women (N=7512, 1279 with hypertension) | 1.38 (1.31,1.45) | 1.33 (1.26,1.40) | 1.16 (1.11,1.21) | 1.12 (1.07,1.17) | 1.35 (1.29,1.41) | 1.30 (1.24,1.35) | 1.36 (1.29,1.42) | 1.31 (1.24,1.37) |
| P-value for difference between area | 0.0089 | 0.3369 | <0.001 | <0.001 | <0.001 | 0.0044 | <0.001 | 0.0015 |

CI: confidence interval; zBMI: z-score, age-standardised body mass index; zWHR: z-score, age-standardised waist-to-hip ratio; zWC: z-score, age-standardised waist-circumference; zWHtR: z-score, age-standardised waist-to-height ratio; *adjusted for smoking status, alcohol intake, wealth, and physical activity level. Diabetes was defined as having any of the following: fasting glucose ≥7.0 mmol/L currently taking antidiabetic medication, or a self-report of a previous diabetes diagnosis. Hypertension was defined as having either a SBP ≥140mmHg or a DBP ≥90mmHg or were on antihypertensive medication.

# SFigures

**SFig. 1**

Mean fasting glucose per fifth of zWC and zWHtR in participants without diabetes (N=22906); zWC: z-score, age-standardised waist-circumference; zWHtR: z-score, age-standardised waist-to-height ratio. Dots/triangles and first numerical results are mean fasting glucose (mmol/l); ends of vertical lines and numerical results in brackets represent the 95% confidence interval of the means

**SFig. 2**

Mean systolic blood pressure per fifth of zWC and zWHtR in participants without hypertension (N=26682); zWC: z-score, age-standardised waist-circumference; zWHtR: z-score, age-standardised waist-to-height ratio. Dots/triangles and first numerical results are mean systolic blood pressure (mmHg); ends of vertical lines and numerical results in brackets represent the 95% confidence interval of the means

**SFig. 3**

Mean diastolic blood pressure per fifth of zWC and zWHtR in participants without hypertension (N=26683); zWC: z-score, age-standardised waist-circumference; zWHtR: z-score, age-standardised waist-to-height ratio. Dots/triangles and first numerical results are mean diastolic blood pressure (mmHg); ends of vertical lines and numerical results in brackets represent the 95% confidence interval of the means

**SFig. 4**

Percent with diabetes per fifth of zWC and zWHtR (N=23148); zWC: z-score, age-standardised waist-circumference; zWHtR: z-score, age-standardised waist-to-height ratio. Dots/triangles and first numerical results are percent with diabetes; ends of vertical lines and numerical results in brackets represent the 95% confidence interval of the percentages. Diabetes was defined as having any of the following: fasting glucose ≥7.0 mmol/L currently taking antidiabetic medication, or a self-report of a previous diabetes diagnosis.

**SFig. 5**

Percent with hypertension per fifth of zWC and zWHtR (N=27880); zWC: z-score, age-standardised waist-circumference; zWHtR: z-score, age-standardised waist-to-height ratio. Dots/triangles and first numerical results are percent with hypertension; ends of vertical lines and numerical results in brackets represent the 95% confidence interval of the percentages. Hypertension was defined as having either a SBP ≥140mmHg or a DBP ≥90mmHg or were on antihypertensive medication.
