# Supplementary figures and images for: How does the association of general and central adiposity with glycaemia and blood pressure differ by gender and area of residence in a Malawian population: a cross-sectional study
Source: Int J Epidemiol. 2018 Apr 10;47(3):887–98. doi: 10.1093/ije/dyy047 (PMC6005143; doi:10.1093/ije/dyy047)

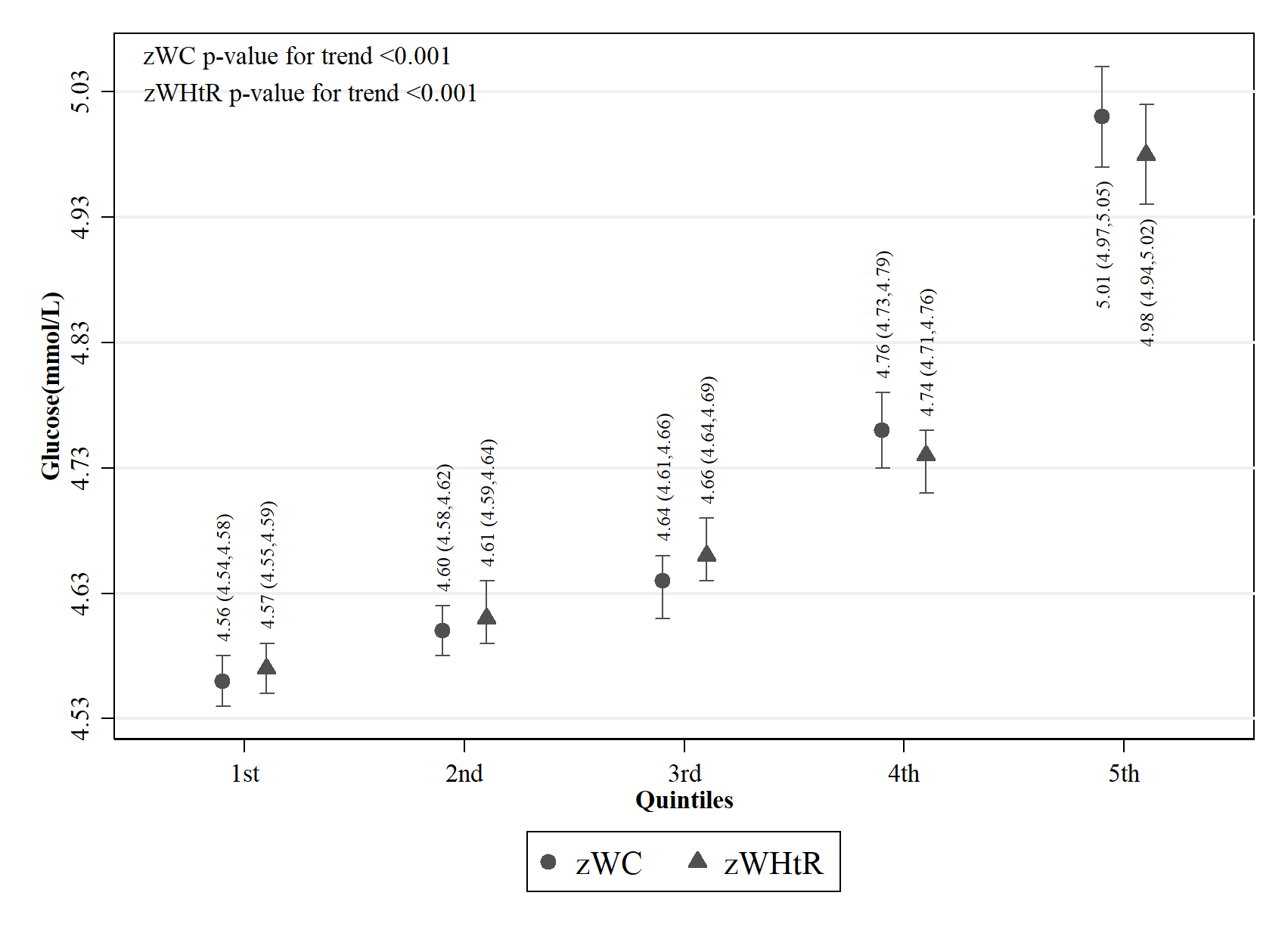

Supplement: Supplementary Data [file dyy047_supp.zip › dyy047-suppl_data/ije-2017-06-0688-File013.tif]

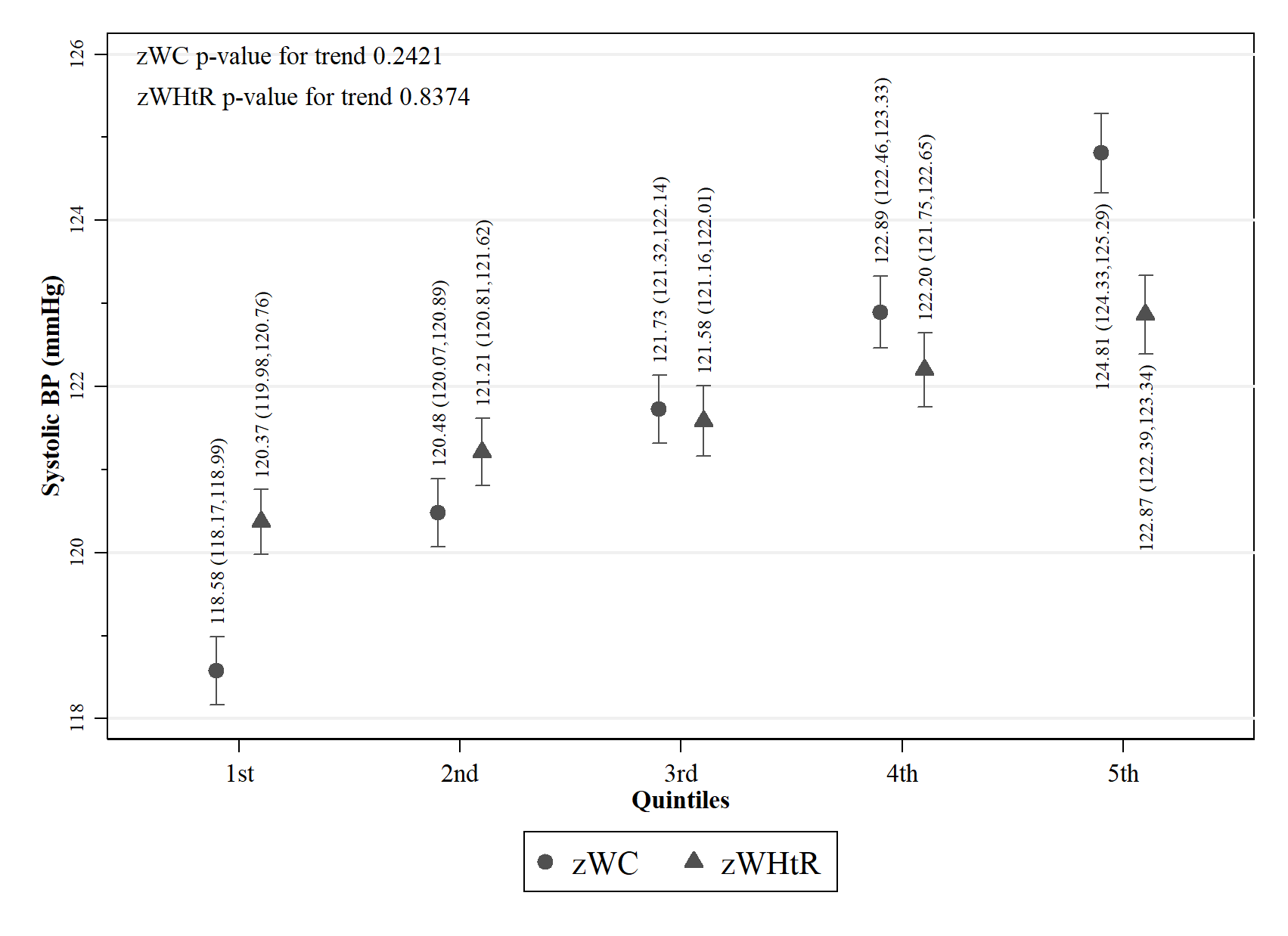

Supplement: Supplementary Data [file dyy047_supp.zip › dyy047-suppl_data/ije-2017-06-0688-File014.tif]

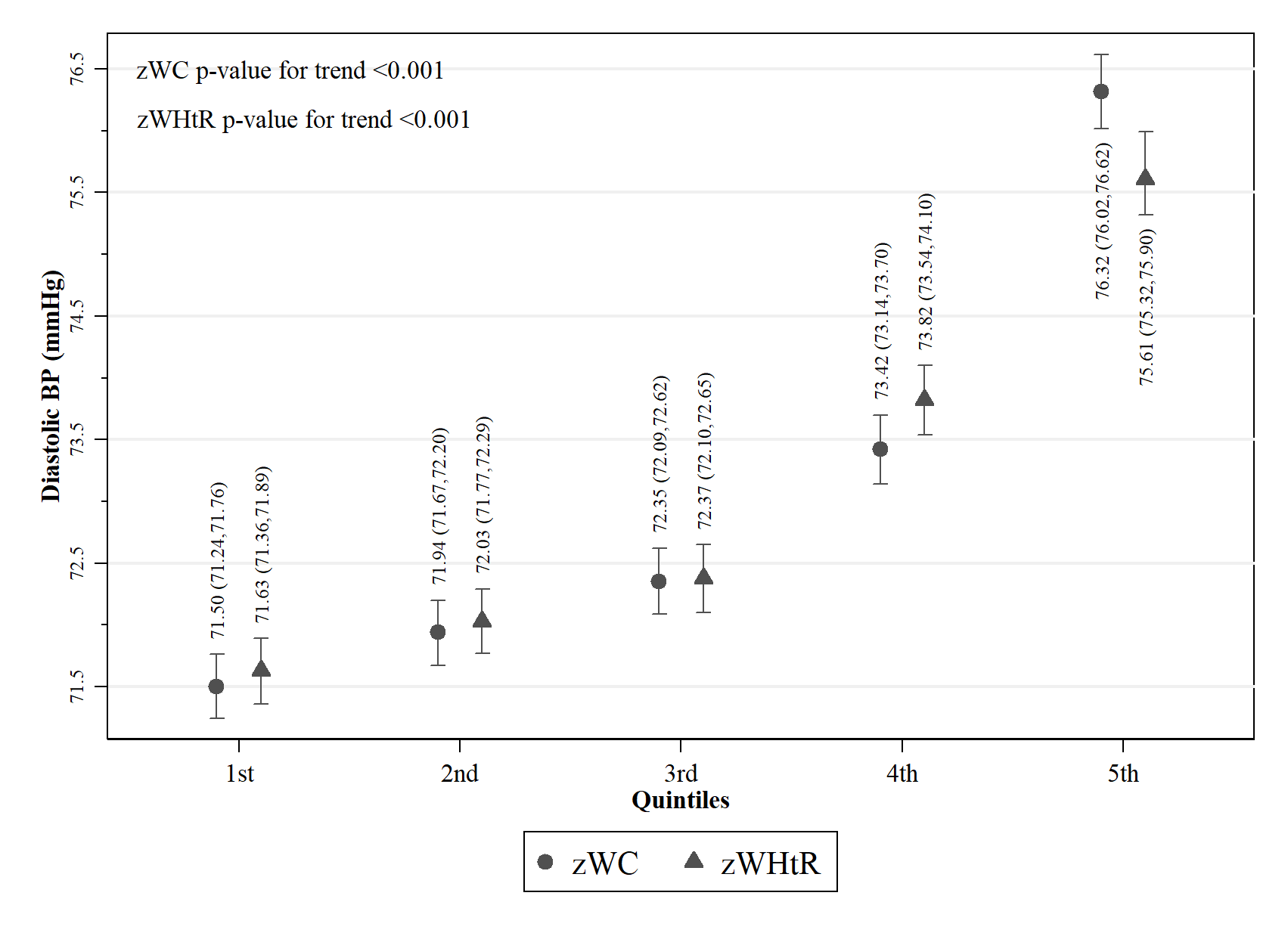

Supplement: Supplementary Data [file dyy047_supp.zip › dyy047-suppl_data/ije-2017-06-0688-File015.tif]

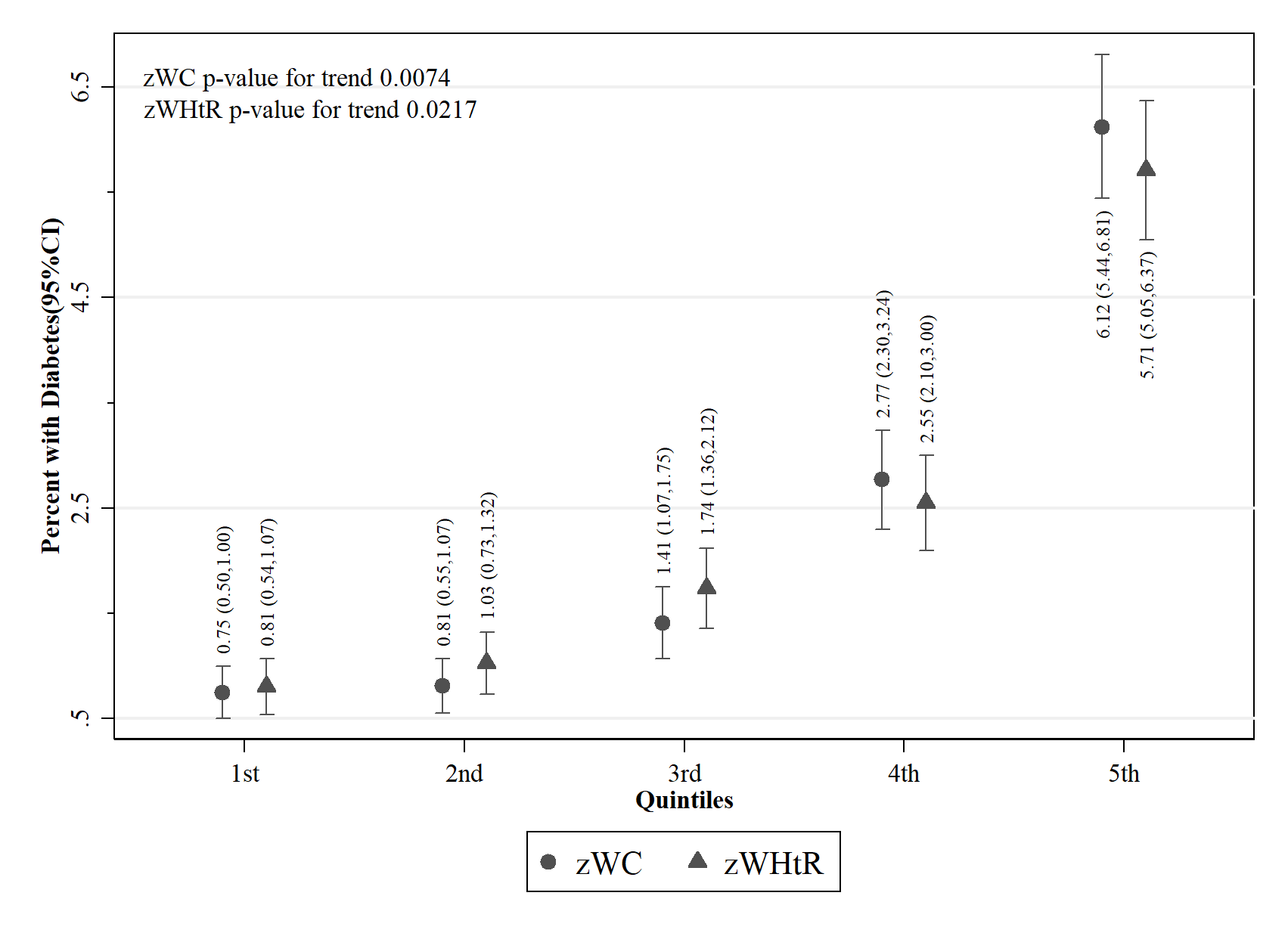

Supplement: Supplementary Data [file dyy047_supp.zip › dyy047-suppl_data/ije-2017-06-0688-File016.tif]

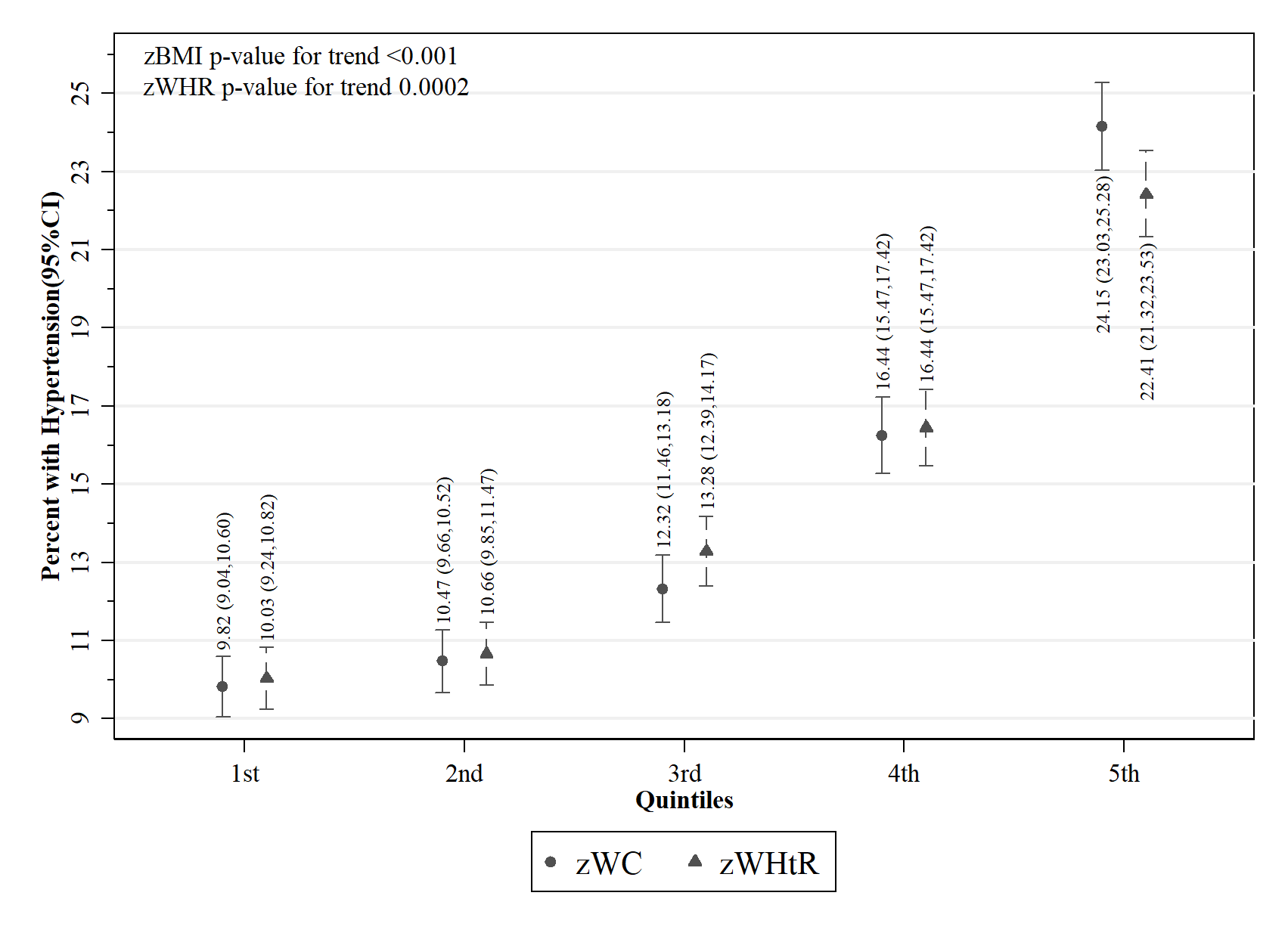

Supplement: Supplementary Data [file dyy047_supp.zip › dyy047-suppl_data/ije-2017-06-0688-File017.tif]
